# Race‐Sex Differences in Statin Use and Low‐Density Lipoprotein Cholesterol Control Among People With Diabetes Mellitus in the Reasons for Geographic and Racial Differences in Stroke Study

**DOI:** 10.1161/JAHA.116.004264

**Published:** 2017-05-10

**Authors:** Christopher M. Gamboa, Lisandro D. Colantonio, Todd M. Brown, April P. Carson, Monika M. Safford

**Affiliations:** ^1^ Division of Preventive Medicine School of Medicine University of Alabama at Birmingham AL; ^2^ Division of Cardiovascular Disease School of Medicine University of Alabama at Birmingham AL; ^3^ Department of Epidemiology School of Public Health University of Alabama at Birmingham AL; ^4^ Weill Cornell Medical College Weill Cornell Medicine New York NY; ^5^ Division of General Internal Medicine Department of Medicine Weill Cornell Medicine New York NY

**Keywords:** diabetes mellitus, gender disparities, low‐density lipoprotein cholesterol, race and ethnicity, statin, Lipids and Cholesterol, Diabetes, Type 2, Race and Ethnicity, Epidemiology, Health Services

## Abstract

**Background:**

Statin therapy is a cornerstone of cardiovascular disease risk reduction for people with diabetes mellitus. Past reports have shown race‐sex differences in statin use in general populations, but statin patterns by race and sex in those with diabetes mellitus have not been thoroughly studied.

**Methods and Results:**

Our sample of 4288 adults ≥45 years of age with diagnosed diabetes mellitus who had low‐density lipoprotein cholesterol (LDL‐C) >100 mg/dL or were taking statins recruited for the Reasons for Geographic and Racial Differences in Stroke study from 2003 to 2007. Exposures included race‐sex groups (white men [WM], black men [BM], white women [WW], black women [BW]) and factors that may influence healthcare utilization. Proportions and prevalence ratios were calculated for statin use and LDL‐C control. Statin use for WM, BM, WW, and BW was 66.0%, 57.8%, 55.0%, and 53.6%, respectively (*P*<0.001). After adjustment for healthcare utilization factors, statin use was lower for BM, WW, and BW compared with WM (prevalence ratios [95%CI]: 0.96 [0.89‐1.03], 0.86 [0.80‐0.92], and 0.87 [0.81‐0.93], respectively, *P*<0.001). LDL‐C control among those taking statins for WM, BM, WW, and BW was 75.3%, 62.7%, 69.0%, and 56.0%, respectively (*P*<0.001). After adjustment, LDL‐C control was lower for BM, WW, and BW compared with WM (prevalence ratios [95%CI]: 0.85 [0.79‐0.93], 0.89 [0.82‐0.96], and 0.73 [0.67‐0.80], respectively, *P*<0.001).

**Conclusions:**

Race‐sex disparities in statin use and LDL‐C control were only partly explained by factors influencing health services utilization. Healthcare provider awareness of these disparities may help to close the observed race‐sex gaps in statin use and LDL‐C control among people with diabetes mellitus.

## Introduction

Individuals with diabetes mellitus are at high risk of coronary heart disease (CHD), and when they experience a clinical CHD event they have a worse prognosis than individuals without diabetes mellitus.^1^, ^2^, ^3^, ^4^, ^5^ Therefore, prevention of CHD is essential for this population.^1^, ^3^, ^5^, ^6^ Major risk factors including dyslipidemia are common among individuals with diabetes mellitus.^7^ Several clinical trials have reported significant CHD risk reduction with statins among individuals with diabetes mellitus both with and without CHD.^1^, ^8^ For these reasons, cholesterol management guidelines have strongly recommended statin therapy among individuals with diabetes mellitus. The 2013 American College of Cardiology (ACC)/American Heart Association Guidelines on the Treatment of Blood Cholesterol to Reduce Atherosclerotic Cardiovascular Risk in Adults recommended statin use for all persons with diabetes mellitus ages 40 to 75 and with low‐density lipoprotein cholesterol (LDL‐C) 70 to 189 mg/dL.^9^ Relevant to the current study, guidelines from the National Cholesterol Education Program's Third Adult Treatment Panel and the American Diabetes Association (ADA) recommended strong consideration of statin therapy for individuals with diabetes mellitus who have a history of CHD or LDL‐C≥100 mg/dL.^1^, ^10^

Although statin use has increased in the United States since 2003,^7^, ^11^ studies have reported underutilization of the medication^12^ and suboptimal LDL‐C control in persons with diabetes mellitus.^7^, ^13^ Lower statin use by blacks and women has been observed in general primary‐care populations,^14^, ^15^, ^16^, ^17^, ^18^, ^19^ but no studies have examined patterns of statin use by race‐sex groups among individuals with diabetes mellitus in the United States. Knowing whether there are race‐sex differences in statin use among adults with diabetes mellitus and understanding the root causes of these disparities are important to design tailored interventions to improve adherence to statin therapy. Therefore, the objectives of this study were (1) to describe statin use patterns and LDL‐C control among black and white men and women from the national Reasons for Geographic and Racial Differences in Stroke (REGARDS) Study who had diabetes mellitus and indication for statin therapy, and (2) to examine whether individual‐level factors known to influence healthcare utilization explain race‐sex differences in statin use and LDL‐C control.

## Methods and Statistics

The REGARDS study has been described previously.^20^ In brief, this population‐based cohort included 30 239 community‐dwelling adults 45 years and older from the 48 contiguous US states and the District of Columbia enrolled between January 2003 and October 2007. The sample was designed to balance sex and black and white race, with oversampling from regions in the Southeastern United States with high stroke mortality referred to as the “stroke buckle” (coastal North Carolina, South Carolina, and Georgia) and the “stroke belt” (the remainder of North Carolina, South Carolina, and Georgia as well as Alabama, Mississippi, Tennessee, Arkansas, and Louisiana). The final cohort included 55% women, 42% blacks, and 55% in the stroke buckle or belt. Data were collected at baseline by computer‐assisted telephone interview and during an in‐home examination by trained health professionals following standardized protocols. The REGARDS study protocol was approved by the Institutional Review Boards governing research in human subjects at the participating centers, and all participants provided written informed consent.

**Figure 1:**
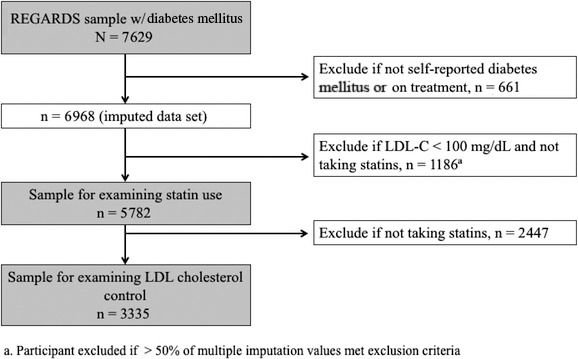
Exclusion cascade describing the 2 study samples. We restricted the 2 study samples to participants who were likely aware that they had diabetes mellitus and therefore would be more likely to be receiving medical care. In addition, we excluded participants missing LDL‐C or if the value was unreliable but maintained them in the sample if they were already taking statins, given that this was the main outcome. An unreliable LDL‐C was defined as triglycerides >400 mg/dL or the participant did not fast. Next, we excluded participants if they were not indicated for statins according to LDL‐C criterion and current statin use. After the first sample examining statin use was defined, we further restricted the sample to only participants taking statins in order to evaluate LDL‐C control among those receiving guideline‐concordant statin therapy. LDL‐C indicates low‐density lipoprotein cholesterol.

The current cross‐sectional analysis used baseline REGARDS data. Of 7629 participants with diabetes mellitus, we selected participants who reported having been told by a healthcare professional that they had diabetes mellitus or who reported taking diabetes mellitus medications (oral agents or insulin) (n=6968; see Figure 1 for a flow diagram of sample construction). There were 661 participants who had diabetes mellitus by glucose criteria alone, defined as fasting glucose ≥126 mg/dL or nonfasting glucose ≥200 mg/dL. Participants with an unreliable or missing LDL‐C (participant did not fast or triglycerides >400 mg/dL) and also not taking statins (n=1800) were excluded. Participants with LDL‐C <100 mg/dL and not on a statin (n=880) were also excluded because contemporary guidelines at the time of enrollment in REGARDS (ie, National Cholesterol Education Program's Third Adult Treatment Panel and ADA guidelines) left consideration of treatment with lipid‐lowering medication at this LDL‐C level to the discretion of the physician and the patient.^1^, ^10^, ^21^ The analytic sample thus included 4288 participants. The analytic sample for examining LDL‐C control restricted this sample to 2482 statin users.

The exposure was self‐reported race‐sex group (white men [WM], black men [BM], white women [WW], and black women [BW]). The outcomes were (1) statin use among the study sample, and (2) LDL‐C control, defined as LDL‐C<100 mg/dL among those taking statins. Statin use was determined through the medication inventory conducted during the in‐home visit, which included all medications taken in the last 2 weeks. LDL‐C was estimated using the Friedewald equation for participants with serum triglycerides <400 mg/dL.^22^

**Figure 2:**
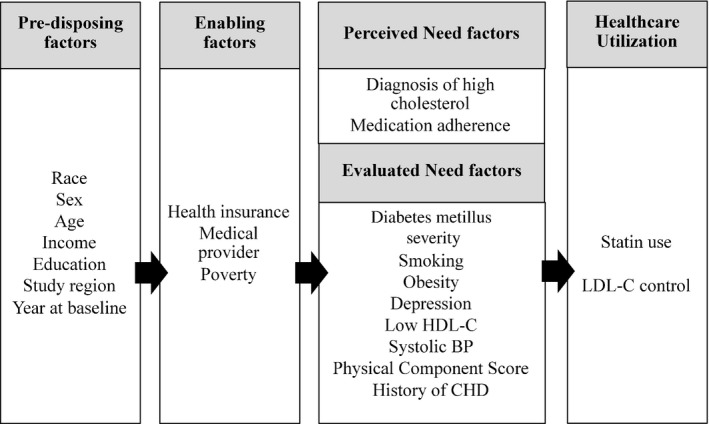
Model of healthcare utilization based on the Andersen and Aday conceptual framework^23^ and adapted to the study of race‐sex differences in statin utilization and LDL‐C control among REGARDS participants with diabetes mellitus and high cholesterol. This conceptual model shows that predisposing factors influence enabling factors, which in turn influence perceived and evaluated need factors. Ultimately, all of the factors affect healthcare utilization and outcomes, defined as statin use and LDL‐C control, respectively. LDL‐C indicates low‐density lipoprotein cholesterol; REGARDS, REasons for Geographic And Racial Differences in Stroke.

We examined associations between race‐sex groups and statin use guided by the Andersen and Aday model of influences on healthcare utilization (Figure 2).^23^ Factors influencing healthcare utilization included predisposing, enabling, and need factors.

Predisposing factors included age, annual household income, highest education level attained (<high school graduation versus ≥graduation), and region of residence (stroke belt or buckle or neither).

Enabling factors included health insurance (insured versus not), having a usual source of medical care (clinic or doctor versus not), and area‐level poverty (defined by the percent of households in the participant's US Census tract living below the US federal poverty line in the year 2000, categorized into tertiles of <9.6%, 9.6% to <21.2%, and ≥21.2%).^24^

Need factors were divided into perceived need and evaluated need. Perceived need was reflected in report of having been told by a doctor they had high cholesterol and medication adherence assessed via the 4‐item Morisky Medication Adherence Scale.^25^ Evaluated need was reflected in diabetes mellitus severity (categorized as using insulin, using oral medications without insulin, or diet controlled; diabetes mellitus medication use was determined through the medication inventory or by self‐report); cigarette smoking (currently smoking versus not); obesity (BMI ≥30 kg/m^2^ based on in‐home measures of height and weight); depressive symptoms (as indicated by a score of 4 or higher on the 4‐item Center for Epidemiology Studies Depression scale)^26^; high‐density lipoprotein cholesterol, considered low if <50 mg/dL for women and <40 mg/dL for men^1^ and dichotomized as low versus not); systolic blood pressure (the average of 2 measurements taken after a seated 5‐minute rest with both feet on the floor and used as a continuous measure); functional status (using the physical component summary scale of the Short Form‐12 used as a continuous measure)^27^; and history of CHD (self‐reported history of myocardial infarction, coronary revascularization [coronary angioplasty or bypass surgery] or electrocardiogram evidence of a prior myocardial infarction [defined as pathological Q‐waves in 2 or more continuous electrocardiogram leads using the Minnesota code]).^28^, ^29^

### Statistical Methods

Characteristics of participants were compared across race‐sex groups. The distributions of continuous variables were checked for normality, and means and standard deviations were reported. Unadjusted tests of association between race‐sex groups and other characteristics were performed and *P*‐values reported using analysis of variance for continuous variables and chi‐squared tests for categorical variables. Prevalence of statin use and LDL‐C control among statin users were calculated by race‐sex group, and *P*‐values from chi‐squared tests were reported. Proportions of participants with statin use and controlled LDL‐C, statin use and uncontrolled LDL‐C, and no statin use and uncontrolled LDL‐C were also calculated by race‐sex group.

Prevalence ratios of statin use among BM, WW, and BW compared to WM were then calculated using Poisson regression with a robust variance estimator. Four incrementally adjusted models were constructed with race‐sex group as the main exposure and statin use as the dependent variable. Model 1 minimally adjusted for age, model 2 added the remaining predisposing factors, model 3 added enabling factors, and model 4 added perceived and evaluated need factors. LDL‐C was not adjusted for in models of statin use because we did not have pretreatment LDL‐C values for those taking statins. Models were repeated using LDL‐C control as the dependent variable among participants treated with statins. The Wald chi‐squared test was used to jointly test for equality of parameter estimates for BM, WW, and BW, and *P*‐values were reported. The level of statistical significance for all tests of association was ∝=0.05.

Poisson regression models were fitted using multiply imputed data. Missing information was imputed by chained equations with m=30 imputations and 10 iterations.^30^ Missing information largely stemmed from income (9%), poverty (7%), medication adherence (3%), and physical component score (3%) alone. The fraction of the sample with any missing information was 31%. All covariates that were adjusted for in models and the outcome were included in the imputation model. Analyses with imputed data were performed in Stata 12 (Stata Corp, College Station, TX), and other analyses were performed in SAS 9.3 (SAS Institute, Cary, NC).

## Results

Baseline characteristics across race‐sex groups of the 4288 participants in the analytic sample are shown in Table 1. WM were slightly older and had higher income and education levels than the other race‐sex groups. Black participants were more likely to live in a higher‐poverty area than white participants. BM were the least likely to have a usual source of medical care and to report having been diagnosed with high cholesterol, whereas WW were the most likely. Diabetes mellitus severity was higher among black participants. Obesity was more prevalent among women and greatest among BW. Women also had higher prevalence of depressive symptoms as well as lower physical functioning compared with men. Men had a higher prevalence of history of CHD, especially WM. White participants had a lower prevalence of low high‐density lipoprotein cholesterol. LDL‐C was calculated, and BW had the highest mean of 117.6 mg/dL (SD=38.6 mg/dL), followed by BM (111.4±35.3 mg/dL), WW (108.9±35.7 mg/dL), and WM (99.3±31.0 mg/dL).

**Table 1:** Baseline Characteristics of REGARDS Participants With Diagnosed Diabetes Mellitus and Either LDL‐C >100 mg/dL or Taking Statins, n=4288

	White Men (n=1089)	Black Men (n=883)	White Women (n=827)	Black Women (n=1489)	*P* Value
Predisposing factors					
Age, mean±SD, y	66.9±8.4	65.4±8.6	65.0±9.2	64.6±8.7	<0.001
Household income, n (%)					<0.001
≥$75 000	247 (18.5)	122 (11.7)	91 (9.1)	66 (3.7)	
$35 000 to $74 000	491 (36.8)	299 (28.8)	240 (23.9)	283 (16.0)	
$20 000 to $34 000	328 (24.6)	293 (28.2)	287 (28.6)	463 (26.2)	
<$20 000	141 (10.6)	215 (20.7)	252 (25.1)	656 (37.2)	
Not disclosed	126 (9.5)	110 (10.6)	135 (13.4)	296 (16.8)	
Less than high school education, n (%)	119 (8.9)	229 (22.1)	114 (11.4)	469 (26.7)	
Stroke region, n (%)					<0.001
Nonbelt	549 (41.2)	543 (52.3)	337 (33.5)	746 (42.3)	
Belt	489 (36.7)	299 (28.8)	391 (38.9)	617 (35.0)	
Buckle	295 (22.1)	197 (19.0)	277 (27.6)	401 (22.7)	
Year at data collection, n (%)					<0.001
2003	335 (25.1)	238 (22.9)	80 (8.0)	221 (12.5)	
2004	468 (35.1)	395 (38.0)	259 (25.8)	493 (27.9)	
2005	216 (16.2)	157 (15.1)	281 (28.0)	447 (25.3)	
2006	146 (11.0)	127 (12.2)	219 (21.8)	342 (19.4)	
2007	168 (12.6)	122 (11.7)	166 (16.5)	261 (14.8)	
Enabling factors					
Has health insurance, n (%)	1292 (97.0)	958 (92.5)	950 (94.5)	1590 (90.2)	<0.001
Has regular source of medical care, n (%)	838 (81.9)	569 (73.2)	667 (85.9)	1126 (82.6)	<0.001
Census tract poverty tertile, n (%)					<0.001
Least poverty	517 (43.6)	147 (15.2)	350 (39.8)	173 (10.6)	
Intermediate poverty	420 (35.4)	323 (33.4)	328 (37.3)	459 (28.2)	
Most poverty	249 (21.0)	496 (51.3)	202 (23.0)	993 (61.1)	
Perceived need factors					
Recall of a diagnosis of high cholesterol, n (%)	984 (74.3)	690 (66.9)	798 (79.6)	1283 (73.3)	<0.001
Perfect medication adherence, n (%)	853 (66.1)	704 (70.8)	638 (65.7)	1167 (68.8)	0.04
Evaluated need factors					
Diabetes mellitus severity, n (%)					<0.001
Diet‐controlled only	248 (23.3)	110 (12.7)	219 (27.2)	230 (15.6)	
Oral medication use only	626 (58.9)	510 (59.0)	447 (55.5)	842 (57.0)	
Any insulin use	189 (17.8)	244 (28.2)	139 (17.3)	404 (27.4)	
Current smoking, n (%)	121 (11.1)	123 (14.0)	116 (14.1)	211 (14.2)	0.02
Obesity, n (%)	519 (48.0)	436 (50.0)	479 (58.5)	991 (67.1)	<0.001
Depressive symptoms, n (%)	104 (9.6)	104 (11.9)	142 (17.3)	301 (20.3)	<0.001
Low HDL‐C, n (%)^a^	527 (48.4)	571 (64.7)	372 (45.0)	804 (54.0)	<0.001
SBP, mean±SD	128.9±16.3	134.6±17.1	126.9±15.8	132.8±17.2	0.002
PCS, mean±SD	44.9±10.8	44.1±10.1	40.6±12.2	40.9±11.4	<0.001
History of CHD, n (%)	458 (42.1)	230 (26.0)	169 (20.4)	284 (19.1)	<0.001

CHD indicates coronary heart disease; HDL‐C, high‐density lipoprotein cholesterol; LDL‐C, low‐density lipoprotein cholesterol; PCS, physical component score; REGARDS, REasons for Geographic And Racial Differences in Stroke; SBP, systolic blood pressure.

a Low HDL‐C defined as <50 mg/dL for women or <40 mg/dL for men.

In this sample, 57.9% of participants were taking statins. Compared with WM, the prevalence of statin use was 8.3, 11.0, and 12.4 percentage points lower for BM, WW, and BW, respectively (Table 2). Among those treated with statins, 65.4% had LDL‐C controlled. Compared with WM, the prevalence of LDL‐C control among the statin‐treated was 12.6, 6.3, and 19.3 percentage points lower for BM, WW, and BW, respectively (Table 2).

**Table 2:** Prevalence and Prevalence Ratios Comparing Statin Use and, Among Those Taking Statins, LDL‐C Control (LDL‐C <100 mg/dL) Across Race‐Sex Groups

	Race‐Sex Group				*P* Value
	White Men	Black Men	White Women	Black Women	
Statin use					
n_statin use_/n	719/1089	510/883	455/827	798/1489	···
% statin use	66.0	57.8	55.0	53.6	<0.001
Models^a^		PR (95%CI)	PR (95%CI)	PR (95%CI)	
1	1 (ref)	0.88 (0.82, 0.95)	0.84 (0.78, 0.91)	0.82 (0.77, 0.88)	<0.001
2	1 (ref)	0.88 (0.82, 0.95)	0.83 (0.76, 0.89)	0.82 (0.76, 0.88)	<0.001
3	1 (ref)	0.89 (0.83, 0.96)	0.82 (0.76, 0.89)	0.82 (0.76, 0.88)	<0.001
4	1 (ref)	0.96 (0.89, 1.03)	0.86 (0.80, 0.92)	0.87 (0.81, 0.93)	<0.001
LDL‐C control, among those taking statins and with valid LDL‐C measurement					
n_LDL‐C controlled_/n	542/719	320/510	314/455	447/798	···
% LDL‐C control	75.3	62.7	69.0	56.0	<0.001
Models^a^		PR (95%CI)	PR (95%CI)	PR (95%CI)	
1	1 (ref)	0.84 (0.78, 0.91)	0.92 (0.86, 0.99)	0.75 (0.70, 0.81)	<0.001
2	1 (ref)	0.85 (0.78, 0.92)	0.87 (0.81, 0.94)	0.72 (0.66, 0.78)	<0.001
3	1 (ref)	0.84 (0.77, 0.91)	0.87 (0.81, 0.94)	0.72 (0.66, 0.78)	<0.001
4	1 (ref)	0.85 (0.79, 0.93)	0.89 (0.82, 0.96)	0.73 (0.67, 0.80)	<0.001

Models are adjusted for healthcare utilization factors. LDL‐C indicates low‐density lipoprotein cholesterol; PR, prevalence ratio.

a Models adjust for healthcare utilization factors as follows: 1—age; 2—model 1+remaining predisposing factors (household income, education, stroke region, year of data collection); 3—model 2+enabling factors (health insurance, medical provider, census tract poverty); 4—model 3+perceived and evaluated need factors (diagnosis of high cholesterol, medication adherence, diabetes mellitus severity, current smoking, obesity, depressive symptoms, high‐density lipoprotein cholesterol, systolic blood pressure, physical component score, and history of coronary heart disease).

**Figure 3:**
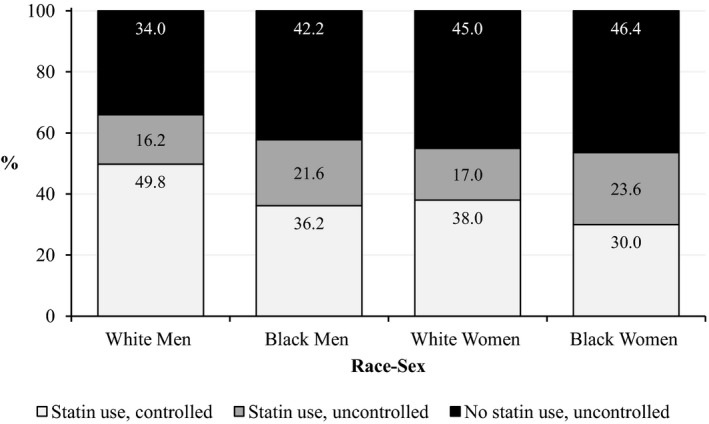
Prevalence of statin use and LDL‐C control by race‐sex group. Results are among participants with diagnosed diabetes mellitus and either LDL‐C >100 mg/dL or taking statins. The 3 categories of statin use and LDL‐C control are mutually exclusive, and percentages in each column sum to 100%. LDL‐C indicates low‐density lipoprotein cholesterol.

When examining statin use and LDL‐C control concurrently, 37.9% of participants were taking statins and had controlled LDL‐C, 20.0% were taking statins and had uncontrolled LDL‐C, and 42.1% were not taking statins and had uncontrolled LDL‐C. Compared with WM, the prevalence of statin use and controlled LDL‐C was 13.6, 11.8, and 19.8 percentage points lower for BM, WW, and BW, respectively (Figure 3). BW had the highest proportion of participants with uncontrolled LDL‐C among both the treated (by 6.6, 2.0, and 7.4 percentage points for WW, BM, and WM, respectively) and the untreated (by 1.4, 4.2, and 12.4 percentage points among WW, BM, and WM, respectively) (Figure 3). Prevalence ratios comparing statin use across race‐sex groups are presented in Table 2. After age adjustment (model 1) and compared with WM, the prevalence of statin use among BM, WW, and BW was lower by 12% (95%CI 5% to 18%), 16% (9% to 22%), and 18% (12% to 23%), respectively. Adjustment for the remaining predisposing factors (model 2), enabling factors (model 3), and need factors (model 4) did not appreciably change these findings. Need factors accounted for some of the race‐sex differences in statin use, but BM, WW, and BW were still, respectively, 4% (95%CI −3% to 11%), 14% (8% to 20%), and 13% (7% to 19%) less likely to be taking statins compared with WM.

Prevalence ratios comparing LDL‐C control among those taking statins across race‐sex groups are also presented in Table 2. After adjustment for age, LDL‐C control was lower in BM, WW, and BW by 16% (95%CI 9% to 22%), 8% (1% to 14%), and 25% (19% to 30%), respectively, compared with WM. Adjustment for the remaining predisposing, enabling, and need factors did not explain these race‐sex differences, and LDL‐C control remained statistically significantly lower in all race‐sex groups compared with WM; BM, WW, and BW were 15% (95%CI 7% to 21%), 11% (4% to 18%), and 27% (20% to 33%) less likely to be controlled.

Associations of predisposing, enabling, and need factors with statin use are presented in Table 3 in a fully adjusted model. The strongest associations with greater statin use were for more severe diabetes mellitus, history of CHD, and health insurance. No recall of high‐cholesterol diagnosis was strongly associated with less statin use. Associations of factors with LDL‐C control are also presented in Table 3. Greater LDL‐C control was most strongly associated with year of data collection, followed by health insurance and more severe diabetes mellitus.

**Table 3:** Prevalence Ratios Comparing Associations Between Healthcare Utilization Factors and Each Outcome: Statin Use and, Among Those Taking Statins, LDL‐C Control (LDL‐C <100 mg/dL)

Factors Influencing Healthcare Utilization	Statin Use	LDL‐C Control
	Fully Adjusted^a^ PR (95%CI)	Fully Adjusted^a^ PR (95%CI)
Predisposing factors		
Race‐sex group		
White men	1 (ref)	1 (ref)
Black men	0.96 (0.89, 1.03)	0.85 (0.79, 0.93)
White women	0.86 (0.80, 0.92)	0.89 (0.82, 0.96)
Black women	0.87 (0.81, 0.93)	0.73 (0.67, 0.80)
Age per SD^b^	1.05 (1.02, 1.08)	1.06 (1.03, 1.10)
Income		
≥$75 000	1 (ref)	1 (ref)
$35 000 to $74 000	1.00 (0.92, 1.08)	1.01 (0.92, 1.11)
$20 000 to $34 000	0.95 (0.87, 1.03)	1.04 (0.94, 1.15)
<$20 000	0.98 (0.89, 1.08)	1.01 (0.90, 1.14)
Less than high school completion vs ≥high school completion	0.99 (0.92, 1.06)	0.98 (0.90, 1.07)
Stroke region		
Nonbelt	1 (ref)	1 (ref)
Belt	0.93 (0.88, 0.98)	1.05 (0.98, 1.12)
Buckle	1.00 (0.94, 1.06)	1.04 (0.96, 1.11)
Year of data collection		
2003	1 (ref)	1 (ref)
2004	1.03 (0.96, 1.11)	1.13 (1.02, 1.25)
2005	1.07 (0.99, 1.16)	1.27 (1.14, 1.40)
2006	1.08 (0.99, 1.17)	1.32 (1.19, 1.47)
2007	1.05 (0.96, 1.14)	1.26 (1.13, 1.41)
Enabling factors		
Health insurance vs no health insurance	1.18 (1.05, 1.33)	1.19 (1.00, 1.41)
Has regular source of medical care vs no regular source	1.00 (0.95, 1.05)	1.01 (0.94, 1.10)
Census tract poverty tertile		
Least poverty	1 (ref)	1 (ref)
Intermediate poverty	0.97 (0.91, 1.03)	1.07 (0.99, 1.15)
Most poverty	0.97 (0.90, 1.04)	1.02 (0.94, 1.11)
Perceived need factor		
No recall vs recall of high cholesterol diagnosis	0.39 (0.35, 0.43)	1.19 (1.11, 1.27)
Imperfect vs perfect medication adherence	1.00 (0.95, 1.05)	0.97 (0.91, 1.03)
Evaluated need factors		
Diabetes mellitus severity		
Diet‐controlled	1 (ref)	1 (ref)
Oral medication use	1.45 (1.33, 1.56)	1.12 (1.02, 1.23)
Insulin use	1.50 (1.37, 1.63)	1.11 (1.00, 1.23)
Current vs not current smoking	0.96 (0.89, 1.03)	1.01 (0.92, 1.10)
Obesity vs no obesity	1.02 (0.97, 1.07)	1.02 (0.96, 1.08)
Depressive vs few/no depressive symptoms	0.93 (0.86, 1.00)	0.97 (0.89, 1.06)
Low vs high HDL‐C	0.98 (0.94, 1.03)	1.06 (1.01, 1.12)
SBP per SD^c^	0.97 (0.95, 0.99)	0.96 (0.93, 0.99)
PCS per SD^d^	0.97 (0.95, 1.00)	0.98 (0.95, 1.01)
History vs no history of CHD	1.22 (1.16, 1.28)	1.03 (0.97, 1.10)

Models are simultaneously adjusted for all healthcare utilization factors. CHD indicates coronary heart disease; HDL‐C, high‐density lipoprotein cholesterol; LDL‐C, low‐density lipoprotein cholesterol; PCS, physical component score; PR, prevalence ratio; REGARDS, REasons for Geographic and Racial Differences in Stroke; SBP, systolic blood pressure.

a Model adjusts for all healthcare utilization factors simultaneously.

b Age standard deviation for statin use sample was 8.8 years; for LDL‐C control sample, 8.4 years.

c SBP standard deviation for statin use sample was 16.9 mm Hg; for LDL‐C control sample, 16.5 mm Hg.

d PCS standard deviation for statin use sample was 11.3 points; for LDL‐C control sample, 11.3 points.

## Discussion

Among these participants with diagnosed diabetes mellitus and evidence of high LDL‐C, a greater proportion of WM were treated with statins compared with BM, BW, and WW. Furthermore, among those taking statins, WM achieved LDL‐C control in greater proportions than all other race‐sex groups, especially compared with BW. Factors proposed to influence health services utilization had little influence on these findings, except for black men, among whom differences compared with white men were modestly explained by factors influencing health services utilization. These results suggest that other factors such as physician prescribing patterns or patient‐provider communication may play an important role in these disparities in statin use. For black men the Andersen and Aday model explained more of the disparity, highlighting the need to better engage black men in their health care.

The finding in this study that WM were more likely than other race‐sex groups to be treated and to have controlled LDL‐C corroborates evidence from several studies. Among US ambulatory patients with diabetes mellitus in 2002‐2004, Segars and Lea found that men were 38% more likely to be given a prescription for a statin, although racial differences were not found.^18^ In an NHANES analysis Mann and colleagues found that, from 1999 to 2004, blacks were 39% less likely than whites to be taking statins.^14^ Additionally, Safford et al reported that among the larger group of REGARDS participants, similar patterns of greater statin treatment and LDL‐C control among the treated were observed for WM compared to the other race‐sex groups.^31^ The current study builds on this past work by demonstrating that the high‐risk group of BM, BW, and WW with diabetes mellitus are at similar risk of undertreatment and uncontrolled LDL‐C.^31^

Despite large race‐sex differences in predisposing factors that were thought to be associated with statin use, adjustment for these only explained 1 percentage point difference in statin use of other race‐sex groups compared to WM; there was no effect on LDL‐C control. Living in the stroke belt was associated with lower statin use, which contrasts Segars and Lea's lack of evidence for regional effects on statin prescriptions.^18^ More recent years were marginally associated with statin use and strongly associated with LDL‐C control, but these temporal trends did not fully account for race‐sex differences in the outcomes. Older age was associated with both outcomes.

Enabling factors did not account for any race‐sex difference in statin use or LDL‐C control. Health insurance was associated with taking statins and LDL‐C control, a finding supported by Mann et al with regard to statin use and by Ali et al in a national study of LDL‐C control improvement.^13^, ^14^ Inadequate insurance coverage itself could have been a barrier to obtaining statins.

Need factors explained a small fraction of race‐sex differences in statin use and LDL‐C control. Not recalling a diagnosis of high cholesterol was an important barrier to statin use for the participants in this study, all of whom were indicated for statins. Possible explanations include lack of a regular source of care or not having LDL‐C measured. Most participants had a regular source of care in this study, however, and adjusting for this characteristic did not explain race‐sex differences in statin use or LDL‐C control. Screening can improve treatment rates,^32^ but we were unable to evaluate the effect of LDL‐C measurement. Differences in recall of high‐cholesterol diagnosis across race‐sex groups could also stem from low healthcare provider recognition or suboptimal communication of hyperlipidemia by providers.^33^ Alternatively, unassessed patient factors in acceptance or reporting of the condition may play a role. Patient‐provider communication is essential in helping patients to understand their condition and overcome emotional reactions that—if not managed well—could lead to denial of illness.^34^, ^35^

A potential explanation for the more aggressive use of statins among WM with diabetes mellitus relative to others may lie in treatment patterns and habits of physicians. In 2004 Mosca et al found that although primary care physicians widely supported the National Cholesterol Education Program's Third Adult Treatment Panel guidelines and CHD risk stratification, fewer than half of physicians implemented tools to calculate risk in routine clinical practice; consistent underestimation of CHD risk was a direct result, especially among women.^36^ By 2008 Persell et al confirmed that using risk factor information alone led to risk underestimation. In addition, clinical decision making often departed from the complex treatment guidelines even given risk factors and estimates.^37^ Furthermore, because most acute CHD cases occur among WM, personal clinical experience and a lack of point‐of‐care risk prediction tools may lead physicians to treat WM more aggressively compared with other race‐sex groups.^38^ The finding in this study that those with a history of CHD and more severe diabetes mellitus were more likely to be treated with statins supports the hypothesis that physicians may be expending efforts on those they perceive to be at highest risk. Current hyperlipidemia treatment guidelines continue to emphasize risk calculation; therefore, interventions such as computerized decision support at the point of care may be warranted.^9^ For example, van Wyk et al showed that a decision support intervention improved treatment from 36% in the control group to 66% in the intervention group.^32^

Strengths of this analysis include the large national, community‐based cohort with oversampling of blacks, which improves generalizability and provides power for race‐sex analyses. A more common approach would be to study race or sex separately, which would have led to different conclusions. The availability of pill bottle review to ascertain statin use and the large number of covariates, including rigorously collected physiologic measures, are other notable strengths that permitted operationalization of several Andersen and Aday model factors. Limitations include that the cross‐sectional, observational design limits the ability to draw causal inferences. Data on health system barriers to statin use such as cost were not available; the availability of $4 drug plans that include statins lessens this concern. Also, we were not able to evaluate some Andersen and Aday domains including health beliefs, perceived discrimination, or family history of hyperlipidemia.

In conclusion, statin use and, among those treated with statins, LDL‐C control were more common among WM compared with other race‐sex groups, even after controlling for cardiovascular disease risk factors and other factors proposed to influence health services utilization. Further study of physician factors that lead to differences in statin prescribing may be warranted.

## Author Contributions

Safford conceptualized the study, and all authors contributed to its design. Gamboa conducted the analyses, and Safford, Colantonio, and Gamboa interpreted the data. Gamboa drafted the manuscript, and all authors reviewed/edited it and have approved its submission.

## Sources of Funding

This research project is supported by a cooperative agreement U01 NS041588 from the National Institute of Neurological Disorders and Stroke, National Institutes of Health, and Department of Health and Human Service (Bethesda, MD). Additional sources of support include grants R01 HL080477 (Safford, Gamboa, Brown, Colantonio) and K24 HL111154 (Safford and Gamboa), both from the National Heart, Lung, and Blood Institute (Bethesda, MD), and grant K01 DK095928 (Carson) from the Agency for Healthcare Research and Quality (Rockville, MD). Representatives of the funding agencies have not been involved in the review of the manuscript and were not directly involved in the collection, management, analysis, or interpretation of the data.

## Disclosures

Dr Safford receives salary support for investigator‐initiated research from Amgen, Inc. Dr Brown participates on observational research with Amgen, Inc, and is the site Principal Investigator for a clinical trial with Omthera Pharmaceuticals. Dr Carson has received salary support for investigator‐initiated research from Amgen, Inc. No other authors reported any disclosures.
